# Reflex‐Mediated Bradycardia and Asystole During Skull Base and Parapharyngeal Surgery: A Three‐Case Series Highlighting Anatomical Predictability

**DOI:** 10.1002/ccr3.73752

**Published:** 2026-10-15

**Authors:** Songyuan Chi, Yiru Wang, Xia Shen

**Affiliations:** ^1^ Department of Anesthesiology, Eye and ENT Hospital Fudan University Shanghai China

**Keywords:** anesthetic management, asystole, bradycardia, cardioinhibitory reflex, cranial neurovascular surgery, skull base surgery

## Abstract

Intraoperative bradycardia and asystole during cranial neurovascular surgery are often regarded as unexpected events. In clinical practice, however, these episodes may occur during manipulation of specific cranial nerve‐rich anatomical regions, suggesting that the risk may be anticipated and clinically actionable. We report three patients who developed suspected reflex‐mediated cardioinhibitory events during surgery involving the jugular foramen, pericarotid skull base, and parapharyngeal space. The events occurred during operations involving regions in which glossopharyngeal or vagal pathways may be vulnerable, although the exact triggering structure could not be confirmed in all cases. Severity ranged from transient bradycardia to prolonged asystole requiring resuscitation. We summarize the cases in a comparative table and propose a practical management pathway emphasizing preoperative recognition of high‐risk anatomical regions, immediate interruption of surgical stimulation, graded pharmacologic treatment, and selective temporary pacing. These cases highlight that reflex‐mediated cardioinhibition in selected skull base and parapharyngeal operations may be anticipated through anatomy‐based perioperative reasoning.

## Key Clinical Message

Bradycardia or asystole during skull base and parapharyngeal surgery may be anticipated when operating near cranial nerve‐rich regions. Immediate cessation of stimulation, exclusion of nonreflex causes, and coordinated escalation by the surgical and anesthetic teams are essential.

## 1 Introduction

Bradycardia, sinus arrest, and asystole during skull base, parapharyngeal, or pericarotid surgery are uncommon but potentially life‐threatening. Such events are often managed as sudden intraoperative crises. However, in operations involving the jugular foramen, cranial nerve rootlets, carotid sheath, and parapharyngeal space, abrupt cardioinhibition may reflect activation of anatomically plausible neurogenic reflex pathways rather than random hemodynamic instability. We present three cases of reflex‐mediated bradycardia or asystole during skull base and parapharyngeal surgery. The purpose of this report is to highlight high‐risk anatomical regions, summarize likely reflex mechanisms, and provide practical perioperative management considerations for anesthesiologists and surgical teams.

### 1.1 Case Reports

#### 1.1.1 Case 1

A 54‐year‐old man with recurrent nonkeratinizing nasopharyngeal carcinoma after chemoradiotherapy underwent endoscopic salvage skull base resection after multidisciplinary evaluation for progressive local recurrence and limited nonsurgical options. Preoperative MRI showed tumor involving the left nasopharynx, skull base, deep parapharyngeal tissues, and pericarotid region. Because the tumor involved the left internal carotid artery and posed a substantial risk of major hemorrhage, planned endovascular occlusion of the artery had been performed preoperatively.

General anesthesia was induced uneventfully and maintained with sevoflurane and remifentanil. Recurrent episodes of bradycardia began approximately 2 h after incision, followed by abrupt asystole at approximately 6 h (Figure 1A). Surgical stimulation was stopped. Cardiopulmonary resuscitation was initiated with chest compressions, atropine, and epinephrine, and spontaneous circulation returned after approximately 5 min. The operation was terminated. No hypoxemia, hypercapnia, metabolic abnormality, baseline hypotension, or progressive hemodynamic deterioration preceded either event.

**FIGURE 1:**
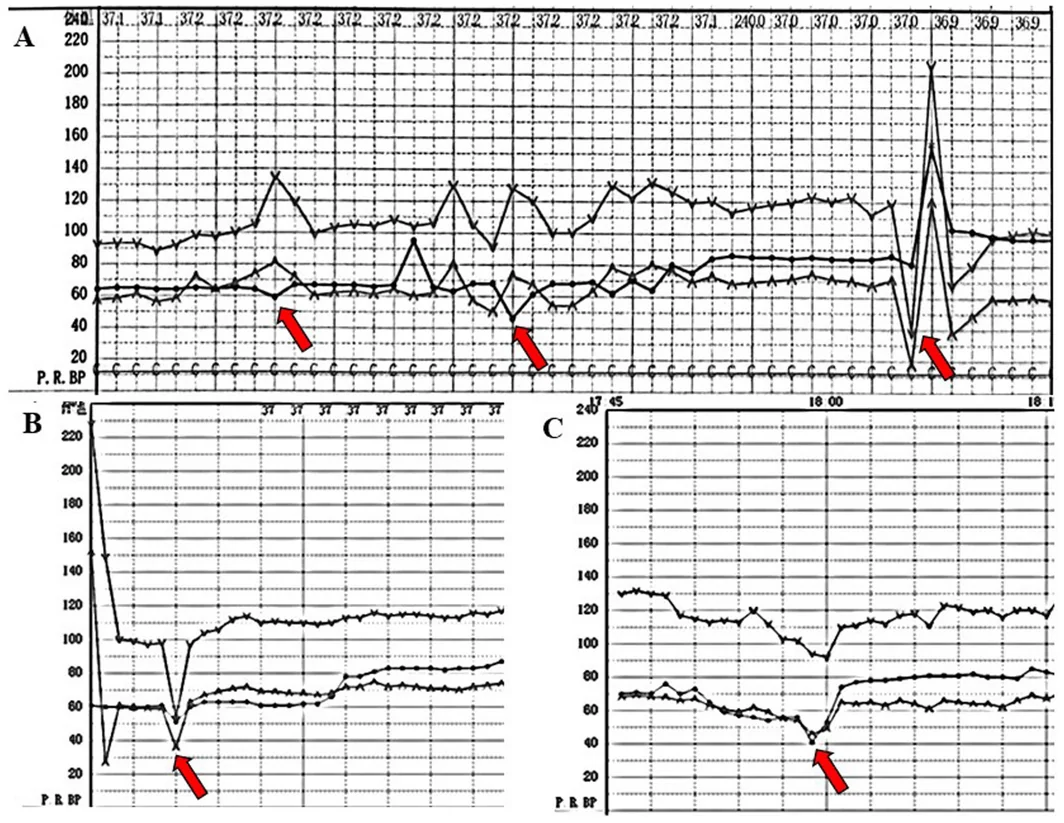
Intraoperative documentation of cardioinhibitory events. (A) Anesthesia record showing recurrent episodes of bradycardia followed by abrupt asystole during surgery. (B) Multiparameter monitor image showing sudden sinus arrest during tumor dissection near the jugular foramen. (C) Monitor replay showing abrupt bradycardia during parapharyngeal tumor manipulation. Arrows indicate the corresponding cardioinhibitory events.

The operative record did not identify the exact structure being manipulated at the onset of either event. With no alternative nonreflex cause identified, a reflex‐mediated mechanism was considered plausible. However, the specific trigger site and neural pathway could not be confirmed. Vagal or glossopharyngeal‐vagal activation therefore remains an anatomy‐based hypothesis (Figure 2A1–A3).

**FIGURE 2:**
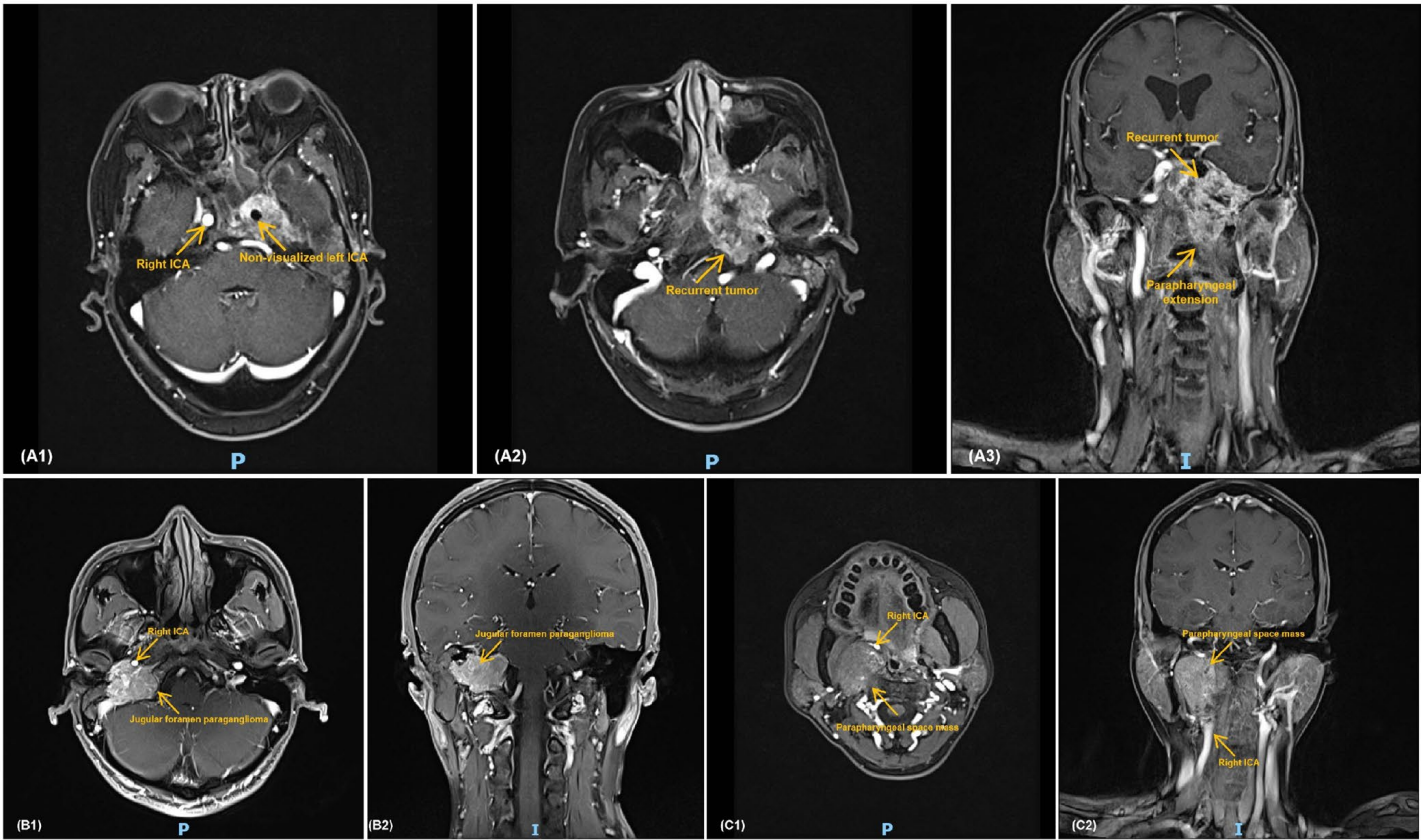
Preoperative MRI and relevant neurovascular anatomy. Representative axial and coronal MRI images show each lesion and selected adjacent vascular structures. (A1–A3) Case 1: Recurrent nasopharyngeal carcinoma involving the left skull base and deep parapharyngeal and pericarotid regions. A1 shows the right internal carotid artery and nonvisualization of the left internal carotid artery; previously planned endovascular occlusion was documented in the medical record. A2 and A3 show the recurrent tumor in axial and coronal planes. The exact structure manipulated at the onset of bradycardia and asystole was not documented. (B1, B2) Case 2: Right jugular foramen paraganglioma centered at the jugular foramen and adjacent to the lower skull base neurovascular structures. (C1, C2) Case 3: Right parapharyngeal/carotid‐space mass with displacement of adjacent carotid sheath structures and a close relationship to the right internal carotid artery.

The patient recovered without neurologic deficit and subsequently underwent an uneventful second operation under temporary cardiac pacing.

#### 1.1.2 Case 2

A 24‐year‐old man underwent resection of a right jugular foramen paraganglioma. Preoperative MRI showed an irregular mass centered at the right jugular foramen, extending around the lower skull base neurovascular structures and closely related to the jugular bulb and internal jugular vein region (Figure 2B1,B2).

During tumor dissection inferior to the internal carotid artery and around the jugular foramen, sudden sinus arrest occurred (Figure 1B). Surgical stimulation was stopped immediately, atropine 0.5 mg was administered intravenously, and brief chest compressions were initiated. Spontaneous cardiac activity returned within approximately 10 s. No hypoxemia, hypercapnia, anesthetic overdose, or metabolic abnormality was identified before the event.

After hemodynamic stabilization, surgery resumed with gentler manipulation, with no further cardioinhibitory episodes. The close temporal association with jugular foramen manipulation was considered consistent with a reflex‐mediated response, most plausibly involving a glossopharyngeal‐vagal pathway. The operation and postoperative course were otherwise uneventful, with no further cardiovascular complications.

#### 1.1.3 Case 3

A 53‐year‐old woman underwent resection of a right parapharyngeal space mass. Preoperative MRI showed an approximately 4.4 × 3.1 × 4.5 cm parapharyngeal/carotid‐space mass, radiologically suspected to be a schwannoma, with displacement of adjacent carotid sheath structures (Figure 2C1,C2).

Bradycardia occurred during tumor mobilization and dissection (Figure 1C). Surgical manipulation was stopped immediately, and the heart rate returned to baseline after atropine 0.3 mg administration. No hypoxemia, hypercapnia, anesthetic overdose, metabolic abnormality, or baseline hypotension preceded the event.

The close temporal association with tumor manipulation and its anatomical relationship to the carotid sheath were considered consistent with a reflex‐mediated cardioinhibitory response, most plausibly involving a vagal pathway. The procedure was completed without further hemodynamic instability, and no recurrent bradyarrhythmia occurred postoperatively.

### 1.2 Differential Considerations

Potential causes of intraoperative bradycardia or asystole included hypoxemia, hypercapnia, anesthetic overdose, electrolyte or metabolic disturbance, hemorrhage‐related instability, myocardial ischemia, and primary conduction disease. In Cases 2 and 3, the events occurred in close temporal association with tumor manipulation in cranial nerve‐rich regions. In Case 1, the events occurred during surgery involving the deep skull base and pericarotid region, although the exact structure being manipulated was not documented. The absence of preceding ventilatory, metabolic, or hemodynamic abnormalities in all three patients supported a reflex‐mediated mechanism as the most plausible explanation.

## 2 Discussion

These cases suggest that cardioinhibitory events may occur during surgery involving cranial nerve‐rich skull base and parapharyngeal regions. In Case 1, tumor involvement of the left internal carotid artery and the associated risk of major hemorrhage prompted planned preoperative endovascular occlusion. During postoperative case review, the surgical team noted that established ICA occlusion also permitted posterior pericarotid dissection. However, the exact structure manipulated at the onset of bradycardia and asystole was not documented, precluding causal attribution. In Cases 2 and 3, the events occurred in close temporal association with tumor manipulation near the jugular foramen and the parapharyngeal/carotid sheath region, respectively. Table 1 summarizes the relevant anatomy, cardioinhibitory events, and management of the three patients.

**TABLE 1:** Summary of relevant anatomy, cardioinhibitory events, and management in the three cases.

Variable	Case 1	Case 2	Case 3
Age/sex	54‐year‐old man	24‐year‐old man	53‐year‐old woman
Diagnosis	Recurrent nonkeratinizing nasopharyngeal carcinoma	Right jugular foramen paraganglioma	Right parapharyngeal/carotid‐space mass, suspected schwannoma
Key imaging/anatomical findings	Left nasopharyngeal and skull base recurrence with deep parapharyngeal and pericarotid extension; left ICA occluded preoperatively	Mass centered at the right jugular foramen, close to lower skull base neurovascular structures	4.4 × 3.1 × 4.5 cm mass displacing carotid sheath structures
Relevant operative region	Deep skull base, parapharyngeal, and pericarotid dissection; exact structure manipulated during the events was not documented	Tumor dissection inferior to the ICA and around the jugular foramen	Tumor mobilization near the carotid sheath region
Cardioinhibitory event	Transient bradycardia followed by abrupt asystole	Sudden sinus arrest	Abrupt bradycardia
Suspected mechanism	Vagal or glossopharyngeal‐vagal reflex	Glossopharyngeal‐vagal reflex	Vagal reflex
Immediate management	Stimulation stopped; CPR, atropine, and epinephrine; operation terminated	Stimulation stopped; atropine 0.5 mg and brief chest compressions; surgery resumed with gentler manipulation	Stimulation stopped; atropine 0.3 mg; surgery continued
Outcome	ROSC after ≈5 min; no neurologic deficit; second operation completed under temporary pacing	Rhythm restored within ≈10 s; no further cardiovascular events	No further instability or postoperative bradyarrhythmia

Abbreviations: CPR, cardiopulmonary resuscitation; ICA, internal carotid artery; ROSC, return of spontaneous circulation.

Similar reflex‐mediated events have been described during skull base, transsphenoidal, and posterior fossa procedures, with trigeminocardiac or glossopharyngeal‐vagal pathways implicated [1, 2]. However, previous reports have largely focused on pathophysiology or isolated cases, whereas generic bradycardia algorithms emphasize treatment after hemodynamic deterioration and seldom incorporate procedure‐specific anatomical risk regions [3, 4]. The present cases therefore highlight the value of anatomy‐based anticipation before entering high‐risk surgical regions.

The presumed mechanisms most likely involved vagal or glossopharyngeal‐vagal activation (Table 2). Afferent input via cranial nerves IX and/or X may be relayed to the nucleus tractus solitarius and subsequently to cardiac vagal motor neurons in the nucleus ambiguus and dorsal motor nucleus of the vagus, leading to parasympathetic activation and cardiac inhibition. Direct vagal activation may also contribute during pericarotid or carotid sheath manipulation, as summarized in Figure S1 [5]. This anatomical framework provides the rationale for coordinated surgical and anesthetic management.

**TABLE 2:** Mechanism‐based differentiation of three intraoperative cardioinhibitory reflex patterns.^a^

Anatomical region/surgical manipulation	Trigger site	Likely afferent‐central‐efferent pathway	Reflex characteristic and clinical implication
Cervical manipulation (carotid sheath/vagal trunk)	Direct mechanical traction on the vagal nerve trunk	Afferent and efferent vagus nerve (cranial nerve X), with relay through medullary vagal nuclei	Peripheral direct vagal reflex; stop traction and treat persistent bradycardia with vagolytic therapy
Jugular foramen/parapharyngeal space	Mechanical stimulation of glossopharyngeal/vagal root fibers	Glossopharyngeal nerve (cranial nerve IX) and vagus nerve (cranial nerve X), relayed through the nucleus tractus solitarius and dorsal motor nucleus of the vagus	Central glossopharyngeal‐vagal reflex; anticipate severe bradycardia or sinus arrest during deep dissection
Carotid sinus	Mechanical or chemical stimulation of carotid sinus receptors	Glossopharyngeal nerve (cranial nerve IX) to nucleus tractus solitarius, followed by vagal efferent activation	Baroreceptor‐mediated reflex; consider gentle manipulation and local afferent modulation when appropriate

^a^ Trigeminocardiac reflexes are not included as no trigeminal‐triggered cases were analyzed in this report.

Management should be organized as a stepwise shared process between the surgical and anesthetic teams, consistent with the need for close interdisciplinary coordination during complex cranial neurovascular surgery [6, 7]. Before entering a high‐risk region, the surgeon should identify the relevant anatomy on preoperative imaging and alert the anesthesiologist before traction, coagulation, or deep dissection. The anesthesiologist should ensure adequate oxygenation, ventilation, and anesthetic depth, with atropine, epinephrine, resuscitation drugs, and pacing or defibrillation equipment readily available.

When bradycardia or sinus arrest occurs, the surgeon should immediately stop stimulation and release traction, while the anesthesiologist excludes hypoxemia, hypercapnia, anesthetic overdose, hemorrhage, and metabolic causes. If bradycardia with a pulse and hemodynamic compromise does not promptly resolve, atropine should be administered. If it remains refractory, an adrenergic agent such as epinephrine or temporary pacing may be considered. Pulseless rhythm or asystole requires immediate chest compressions and advanced life support [3, 8].

After recovery, the decision to continue surgery should be made jointly. If dissection must proceed in the same region, gentler manipulation, local anesthetic infiltration or topical application when anatomically appropriate [9], prophylactic anticholinergic treatment in selected cases [10], and temporary pacing for recurrent or severe bradycardia with a pulse [8, 11] may be considered. A practical management approach is summarized in Figure 3.

**FIGURE 3:**
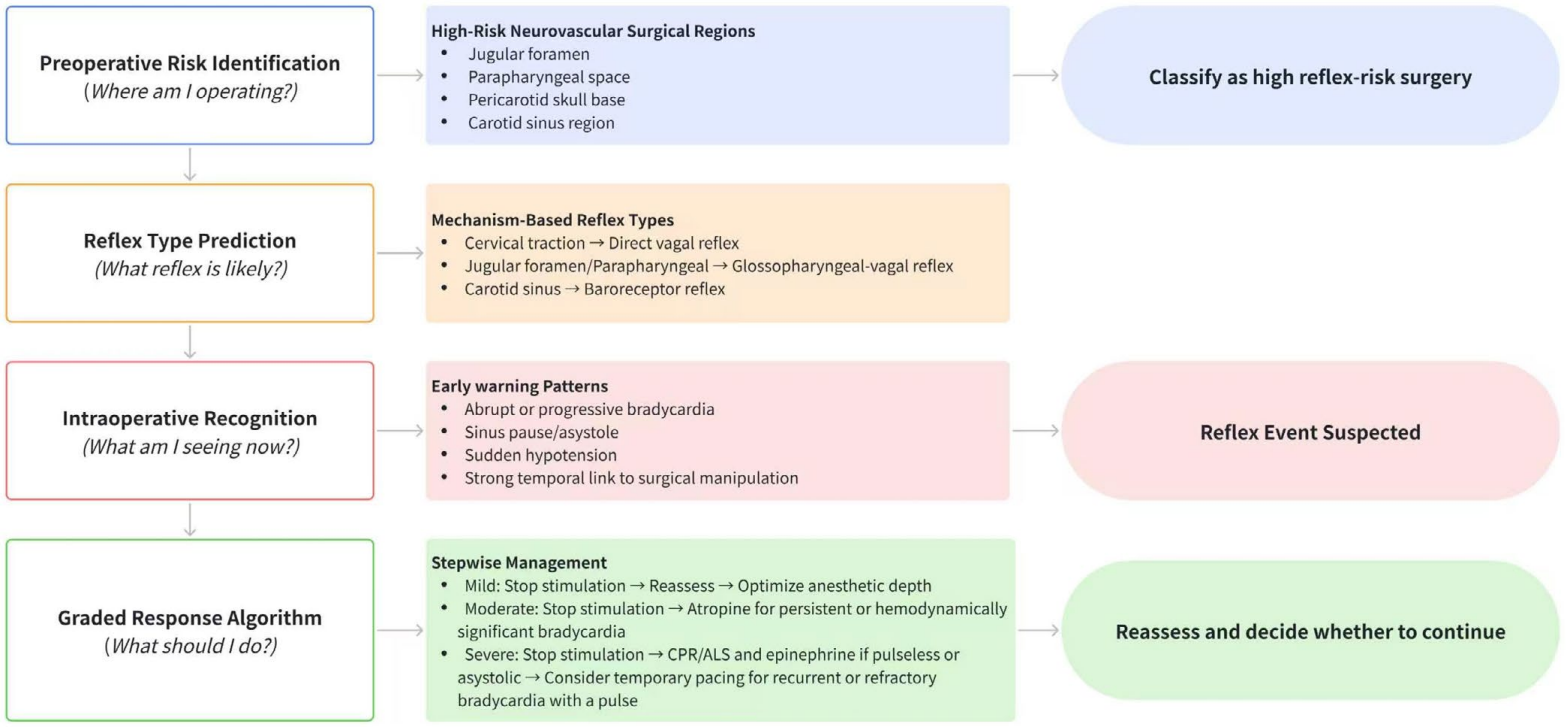
Practical approach to anticipatory recognition and management of reflex‐mediated cardioinhibitory events during cranial neurovascular surgery. The diagram summarizes anatomy‐based risk recognition, immediate interruption of surgical stimulation, graded anesthetic management, and escalation to temporary pacing when required.

In summary, the risk of reflex‐mediated bradycardia and asystole during skull base and parapharyngeal surgery may be anticipated when dissection involves cranial nerve‐rich regions. Preoperative recognition of high‐risk anatomy, immediate cessation of surgical stimulation, and graded team‐based management may reduce progression to severe cardioinhibitory events.

## 3 Limitations

The number of cases is small, and the proposed reflex mechanisms are based on clinical observation, anatomical reasoning, and literature‐supported pathways rather than direct neurophysiologic recording. The exact triggering structure could not be confirmed in all cases. Nevertheless, the overall anatomical and peri‐event findings support reflex‐mediated cardioinhibition as the most plausible explanation and provide clinically useful information for risk anticipation.

## Author Contributions

**Songyuan Chi:** data curation, writing – original draft. **Yiru Wang:** data curation, writing – original draft. **Xia Shen:** conceptualization, supervision.

## Funding

The authors have nothing to report.

## Consent

Written informed consent for publication of anonymized clinical information and intraoperative monitoring images was obtained from each patient included in this case series.

## Conflicts of Interest

The authors declare no conflicts of interest.

## Supporting information


**Figure S1:** Neurophysiological pathway of reflex‐mediated cardioinhibitory responses during cranial neurovascular surgery. The schematic illustrates the reflex arc leading to parasympathetic activation and cardiac inhibition. Trigeminal involvement is shown as a related pathway but was not directly represented in the present cases.

## Data Availability

Research data are not shared.
